# Nerve growth factor induces neurite outgrowth of PC12 cells by promoting Gβγ-microtubule interaction

**DOI:** 10.1186/s12868-014-0132-4

**Published:** 2014-12-31

**Authors:** Jorge A Sierra-Fonseca, Omar Najera, Jessica Martinez-Jurado, Ellen M Walker, Armando Varela-Ramirez, Arshad M Khan, Manuel Miranda, Nazarius S Lamango, Sukla Roychowdhury

**Affiliations:** Neuromodulation Disorders Cluster, Border Biomedical Research Center, University of Texas, El Paso, TX 79968 USA; Cytometry Screening and Imaging Core facility, Border Biomedical Research Center, University of Texas, El Paso, TX 79968 USA; Department of Biological Sciences, University of Texas, El Paso, TX 79968 USA; College of Pharmacy and Pharmaceutical Sciences, Florida A&M University, Tallahassee, FL 32307 USA; Present Address: Department of Pathology, Brigham and Women’s Hospital, Harvard Medical School, Boston, MA 02115 USA

**Keywords:** Neurite outgrowth, Microtubules, Gβγ, Heterotrimeric G proteins, Tubulin

## Abstract

**Background:**

Assembly and disassembly of microtubules (MTs) is critical for neurite outgrowth and differentiation. Evidence suggests that nerve growth factor (NGF) induces neurite outgrowth from PC12 cells by activating the receptor tyrosine kinase, TrkA. G protein-coupled receptors (GPCRs) as well as heterotrimeric G proteins are also involved in regulating neurite outgrowth. However, the possible connection between these pathways and how they might ultimately converge to regulate the assembly and organization of MTs during neurite outgrowth is not well understood.

**Results:**

Here, we report that Gβγ, an important component of the GPCR pathway, is critical for NGF-induced neuronal differentiation of PC12 cells. We have found that NGF promoted the interaction of Gβγ with MTs and stimulated MT assembly. While Gβγ-sequestering peptide GRK2i inhibited neurite formation, disrupted MTs, and induced neurite damage, the Gβγ activator mSIRK stimulated neurite outgrowth, which indicates the involvement of Gβγ in this process. Because we have shown earlier that prenylation and subsequent methylation/demethylation of γ subunits are required for the Gβγ-MTs interaction *in vitro*, small-molecule inhibitors (L-28 and L-23) targeting prenylated methylated protein methyl esterase (PMPMEase) were tested in the current study. We found that these inhibitors disrupted Gβγ and ΜΤ organization and affected cellular morphology and neurite outgrowth. In further support of a role of Gβγ-MT interaction in neuronal differentiation, it was observed that overexpression of Gβγ in PC12 cells induced neurite outgrowth in the absence of added NGF. Moreover, overexpressed Gβγ exhibited a pattern of association with MTs similar to that observed in NGF-differentiated cells.

**Conclusions:**

Altogether, our results demonstrate that βγ subunit of heterotrimeric G proteins play a critical role in neurite outgrowth and differentiation by interacting with MTs and modulating MT rearrangement.

**Electronic supplementary material:**

The online version of this article (doi:10.1186/s12868-014-0132-4) contains supplementary material, which is available to authorized users.

## Background

Neuronal outgrowth is a complex process in which two distinct domains emerge from the cell body: a long, thin axon that transmits signals, and multiple shorter dendrites, which are specialized primarily for receiving signals. When fully differentiated through axon and dendrite elongation, this unique morphology allows neurons to achieve precise connectivity between appropriate sets of neurons, which is crucial for the proper functioning of the nervous system. While many signals are known to drive neuronal outgrowth, it is the assembly and disassembly of cytoskeletal structures embodied within neurite extension and growth cone formation that are essential for establishing appropriate synaptic connections and signal transmission.

Microtubules (MTs) form dense parallel arrays in axons and dendrites that are required for the growth and maintenance of these neurites [1]. Selective stabilization of MTs also occurs during neuronal differentiation [2,3]. In the axon, MTs are bundled by the microtubule-associated protein (MAP) tau, with their plus ends oriented toward the nerve terminal. In contrast, dendritic MTs, bundled instead by MAP2, have a mixed orientation, with their plus ends facing either the dendritic tips or the cell body. Since localized changes in the assembly and organization of MTs are sufficient to alter axon and dendritic specification and development [1], knowledge of the precise signaling mechanisms controlling MT assembly and organization is crucial for our understanding of neuronal plasticity and neurodegenerative diseases.

Over the years, pheochromocytoma (PC12) cells have been used as a model to study neuronal differentiation because they respond to nerve growth factor (NGF) and exhibit a typical phenotype of neuronal cells sending out neurites [4]. NGF is a neurotrophic factor critical for the survival and maintenance of sympathetic and sensory neurons, and it binds to the high-affinity tyrosine kinase receptor, TrkA, leading to its phosphorylation and the subsequent activation of PI3K/Akt/GSK3β pathways. This, in turn, facilitates the cytoskeletal rearrangements necessary for neurite outgrowth [5-8]. The Rho and Ras families of small GTPases are also important regulators of the MTs and the actin cytoskeleton in neurons, and modulate downstream effectors, including serine threonine kinase, p21-activated kinase, ROCK, and mDia [9,10]. The G protein-coupled receptors (GPCRs) and the α and βγ subunits of heterotrimeric G proteins also participate in neurite outgrowth [11-18]. Gβγ has been shown to regulate neurite outgrowth in primary hippocampal neurons by interacting with Tctex-1, a light-chain component of the cytoplasmic dynein motor complex [17]. It has been proposed that Gβγ might accomplish this function by linking extracellular signals to localized regulation of MTs and actin filaments through Rho GTPase and downstream MT modulators [17,19]. PI3K is also a downstream effector of Gβγ in GPCR signaling [20,21], and recent results suggest that the activation of PI3K/Akt pathway by NGF is, in part, mediated through the βγ subunit [19,22,23]. These studies collectively suggest a role of Gβγ in neuronal differentiation. However, the mechanisms by which Gβγ acts to regulate neurite outgrowth are still not well understood.

We have shown earlier that Gβγ binds to tubulin and stimulates MT assembly *in vitro*. Using the MT depolymerizing drug nocodazole, we have demonstrated that Gβγ-MT interaction is critical for MT assembly in cultured PC12 and NIH3T3 cells [24-26]. In the current study, we asked whether Gβγ is involved in NGF-induced neuronal differentiation of PC12 cells through its ability to interact with MTs and modulate MT assembly. We found that the interaction of Gβγ with MTs, and MT assembly increased significantly in response to NGF; and that a Gβγ-sequestering peptide, GRK2i, inhibited neurite outgrowth and induced MT disruption, supporting a critical role of the Gβγ-MT interaction in neurite outgrowth. Furthermore, the overexpression of Gβγ in PC12 cells induced neurite formation in the absence of NGF, and overexpressed protein co-localized with MTs in the neurites. We also found that small-molecule inhibitors of prenylated methylated protein methyl esterase (PMPMEase), an enzyme involved in the prenylation pathway [27], disrupted the MT and Gβγ organization and inhibited neurite outgrowth.

## Methods

### Cell culture and NGF treatment

PC12 cells (pheochromocytoma cells derived from the adrenal gland of *Rattus norvegicus*) (ATCC, Manassas, VA), were grown in 75-cm^2^ culture flasks at 37°C in Dulbecco’s Modified Eagle’s Medium (DMEM) (4.5 g/L glucose, L-glutamine, without pyruvate), supplemented with 10% bovine calf serum and antibiotics (100 U/mL penicillin and 100 μg/mL streptomycin) in 10% CO_2_. For NGF treatment, PC12 cells were treated with 100 ng/mL of NGF (Sigma-Aldrich, St. Louis, MO) dissolved in complete media for three consecutive days. Control cells without NGF were also grown under the same conditions. For quantitative assessment of neurite outgrowth, PC12 cells were only treated with NGF for 2 days instead of 3, given that the density of neurite outgrowth does not allow for proper tracing of neurites belonging to a specific cell body.

### PMPMEase inhibitors (L-28 and L-23), Gβγ-blocking peptide (GRK2i), and Gβγ activator mSIRK

PMPMEase is a key enzyme in the reversible methylation/demethylation step in the protein prenylation pathway. Using phenylmethylsulfonyl fluoride (PMSF) as a prototypical molecule, Aguilar et al. [27] recently synthesized high-affinity-specific inhibitors of PMPMEase. Two such inhibitors, 2-*trans*-Geranylthioethanesulfonyl fluoride (L-23) and 2-*trans, trans*-Farnesylthioethanesulfonyl fluoride (L-28) were used in our study. A stock solution of 20 mM L-23 or L-28 were prepared in DMSO and diluted in tissue culture media to a final concentration of 1, 5, or 10 μM and added to the cells as indicated in the figures. The DMSO concentration in the culture media never exceeded 0.05%. In addition, a control experiment was performed in the presence of a similar concentration of DMSO. The sequence of the Gβγ-blocking peptide GRK2i (WKKELRDAYREAQQLVQRVPKMKNKPRS) from Tocris Bioscience (Bristol, UK), encodes the Gβγ-binding domain of G protein-coupled receptor kinase 2 (GRK2) and acts as a cellular Gβγ antagonist. A stock solution of the peptide (1 mM) was prepared in 10% DMSO, and was added to the cells at final concentrations of 1, 5, or 10 μM as indicated in the figure. The DMSO concentration in the culture media never exceeded 0.1% and as indicated above, the control experiment was also performed in the presence of 0.1% DMSO in the culture media. A stock solution (1 mM) of mSIRK peptide (myr SIRKALNILGYPDYD) (EMD Chemicals) was also prepared similar to that described for GRK-2i.

### Extraction of microtubules (MT) and soluble tubulin (ST) fractions

PC12 cells were grown on 100- or 150-mm plates to 70% confluence over 1–2 days and subjected to NGF and/or various treatments as indicated in the figures. The plates were used in duplicates for each condition. Microtubules (MTs) and soluble tubulin (ST)) fractions were prepared by extracting soluble proteins in an MT stabilizing (MS) buffer as described previously [26] with a minor modification adopted from Marklund et al. [28]. Briefly, cells were rinsed in PBS and incubated with 0.5–1 ml MS buffer (0.1 M PIPES, pH 6.9, 2 M glycerol, 5 mM MgCl2, 2 mM EGTA, 0.5% Triton X-100) containing protease inhibitor cocktail (Roche Applied Science, Indianapolis, IN), 1 mM DTT, and 10 μM GTP) for ~10 min at room temperature. Subsequently, the cells were removed mechanically (using a cell scraper) and centrifuged at 10,000 × g for 10 min. The supernatant constitutes the ST fraction and the cell pellets represent the MT fraction that includes the tubulin polymers. Pellets were washed in PEM buffer (100 mm PIPES, pH 6.9, 2 mM EGTA, 1 mM MgCl2) and resuspended in PEM buffer containing 1 mM DTT, 10 μM GTP, and protease inhibitor cocktail followed by incubation in ice for 30 min. Protein extraction was performed by sonicating the samples on ice for 1 min and clarifying by centrifugation at 10,000 × g for 10 min. This procedure yielded highly reproducible results in technically replicated samples used for each condition in a given experiment.

### Preparation of whole cell lysates

PC12 cells were grown on 100- or 150-mm plates to 80% confluence over 1–2 days. Cells were then treated with or without NGF as indicated. The medium was removed, and the cells were washed with PBS followed by incubating with 0.5–1 mL lysis buffer (10 mM Tris–HCl, pH 7.9, 1.5 mM MgCl2, 0.3 M sucrose, 0.1% Triton X-100, 1 mM DTT, 10 μM GTP, and protease inhibitor cocktail) in ice until the cells were lysed. Cells were then scraped with a rubber policeman and sonicated in ice for 1 min, followed by centrifugation at 10,000 × g for 10 min. Supernatants represent whole-cell lysates. Protein concentrations were typically between 1–2 mg/mL.

### Electrophoresis, immunoblotting, and immunoprecipitation

Samples for immunoblotting were subjected to SDS-polyacrylamide gel (10%) electrophoresis, followed by electrotransfer onto nitrocellulose membranes [29,30]. The membranes were incubated in 5% nonfat dry milk in TBS (10 mM Tris–HCl and 150 mM NaCl, pH 7.4) for 2 h at room temperature, followed by overnight incubation with mouse monoclonal anti-α tubulin [Sigma-Aldrich Cat# T9026 RRID:AB_477593] or rabbit polyclonal anti-Gβ [Santa Cruz Biotechnology Cat# sc-378 RRID:AB_631542] in TBS containing 0.01% BSA (1:200 dilution) as previously described (Montoya et al. [26]). The membranes were washed with 0.05% Tween-20 in TBS (TBST) and incubated with appropriate HRP-conjugated secondary antibodies (goat anti-mouse or goat anti-rabbit from Promega, Madison, WI; 1:1000) in TBST containing 0.01% BSA for 1 h. For sensitive detection, the chemiluminescence (ECL) technique (SuperSignal West Pico Chemiluminescent Substrate) was used according to the manufacturer’s instructions (Pierce Biotechnology, Rockford, IL). Quantitative analysis was done using LabWorks image acquisition and analysis software (UVP Laboratory Products, Upland, CA). For immunoprecipitation, 100 μL aliquots of cellular fractions (~0.25–1 mg/mL) were incubated with or without anti-Gβ, anti-tub (5–10 μl), or non-specific rabbit IgG for 1 h at 4°C, followed by the overnight incubation (4°C) with 100 μL 50% protein A-sepharose (Amersham Biochemical, Piscataway, NJ), as previously described [26]. Samples were then centrifuged at 10,000 × g for 10 min, and the supernatants (SUP) were saved. The pellets (immunocomplex) were washed with TBS and eluted with 3% SDS Laemmli sample buffer containing 0.15 M dithiothreitol (DTT) and boiled in a water bath for 5 min. Samples were then clarified by centrifugation. Both IP and SUP fractions were then subjected to immunoblotting using anti-tubulin or anti-Gβ antibody as discussed above.

### Overexpression of Gβγ

PC12 cells were transiently transfected with yellow fluorescent protein (YFP)-tagged pcDNA3.1 plasmids encoding for Gβ1, Gγ1 or Gγ2 subunits. Cells were either co-transfected with β1 and γ2, β1 and γ1, or transfected with individual constructs (Gβ1, Gγ1, and Gγ2). The expression plasmids were generously provided by Dr. N. Gautam (Washington University, St. Louis, MO). He and his colleagues developed these constructs and showed that the tagged β and γ subunits are functional [31,32]. These constructs are now available through Addgene. A plasmid encoding only YFP (pcDNA3-YFP, Addgene, Cambridge, MA) was used as a control. Cells were transfected with the plasmids using Lipofectamine LTX PLUS reagent (Invitrogen, Carlsbad, CA) according to the manufacturer’s instructions. Briefly, PC12 cells were seeded on glass coverslips using 12-well plates at a density of 50,000 cells/well, and incubated overnight under normal growth conditions. The following day, the cells were transfected with a mixture of Lipofectamine LTX PLUS containing 2 μg of each plasmid (dissolved in antibiotic-free media) and incubated overnight in normal growth media. Cells were monitored for protein expression (YFP fluorescence) and morphological changes using differential interference contrast (DIC) images at different time points (24, 48, and 72 h), using a Zeiss Axiovert 200 fluorescence microscope equipped with a GFP filter. For confocal microscopic analysis, the cells were fixed and processed as described below.

### Confocal microscopy

For confocal microscopic analysis, PC12 cells were allowed to attach to immunocytochemistry slides (Lab-TEK II mounted on glass slides, Thermo Fisher Scientific, Rochester, NY) and were grown overnight as described above. Cells were then treated with or without NGF as indicated and subsequently fixed by the addition of ice-cold 100% methanol (previously cooled to −20°C) and incubated at −20°C for 6 min as described [26]. The cells were then rinsed three times in PBS, blocked for 1 h at room temperature in 5% normal goat serum (NGS) (Sigma-Aldrich) in PBS, followed by overnight incubation at 4°C with mouse monoclonal anti-α tubulin [Sigma-Aldrich Cat# T9026 RRID:AB_477593] and/or rabbit polyclonal anti-Gβ [Santa Cruz Biotechnology Cat# sc-378 RRID:AB_631542] in 1% NGS in PBS (1:100 dilution) as indicated in the figure. The slides were rinsed as before and incubated with the tetramethyl rhodamine (TMR)-conjugated goat anti-mouse IgG and/or fluorescein isothiocyanate (FITC)-conjugated goat anti-rabbit IgG (Molecular Probes-Invitrogen, Carlsbad, CA) for 2 h in the dark to diminish photo-bleaching effects. The slides were then mounted with DAKO mounting media (DAKO Corporation, Carpenteria, CA), or with ProLong Gold anti-fade reagent with DAPI (Invitrogen, Carlsbad, CA) for nuclear staining), and covered with coverslip. High-resolution, digital, fluorescent images were captured by employing inverted, confocal-laser-scanning microscopy (model LSM 700; Zeiss, Thornwood, NY), utilizing a Plan-Apochromat 63×/1.40 immersion-oil DIC objective and assisted with ZEN 2009 software (Zeiss, Thornwood, NY). DAPI (blue), FITC (green), and rhodamine (red) were excited with laser emissions at 405-, 488-, and 555-nm wavelengths, respectively. Gβγ overexpressed cells were only labeled with anti-α-tubulin.

### Co-localization analysis

To quantitatively assess the degree of co-localization between Gβγ and MTs, regions of interest (ROIs) were delimited within cells to decrease the background fluorescence contribution. Co-localization was calculated using a squared Manders’ overlap coefficient of the defined signals, performed on a pixel-by-pixel basis, which represented an accurate degree of co-localization. The overlap coefficient according to Manders provided values within the range from 0 to 1; a value of 0 means that there were no pixels within the selected ROI with overlapped signals, whereas a value of 1 represents perfectly co-localized pixels [33]. The values for selected ROIs were acquired from images taken from 10–12 cells from different microscope fields, using ZEN 2009 software. In order to rule out bleed-through of the fluorescent labels, control coverslips were prepared with a single fluorophore and were further imaged under the same microscope settings used with the double-labeled coverslips.

### 3-D image analysis

Image stacks were imported into Volocity 3-D Image Analysis Software (Version 6.0; Perkin Elmer Corporation, Waltham, MA) operating on a Macintosh Pro computer. In Volocity’s Restoration module, a point-spread function was calculated to deconvolve the native image stack using iterative restoration (80%, 20 iterations max). In Volocity’s Visualization module, a joystick control aided in free flight through the newly rendered 3-D image for selection of proper viewing approaches alongside labeled neurites of the cell. These instances within the moving sequence were bookmarked, and the bookmarks were dropped into the software’s movie-making interface. The final sequence was exported as a QuickTime movie and still frames from this movie sequence were selected to generate.

### Neurite outgrowth assessment

For neurite outgrowth measurement, cells were fixed and processed for confocal microscopy using a mouse monoclonal anti-tubulin antibody and a rabbit polyclonal Gβ antibody, followed by labeling with rhodamine- and FITC-conjugated secondary antibodies. Due to the fast photo-bleaching of the FITC fluorophore, the cells were only imaged using rhodamine staining for the purpose of neurite outgrowth assessment. Cells were viewed using the 40× objective with a Zeiss LSM 700 confocal microscope. The coverslips were scanned from left to right, and 8–10 fields were randomly selected. For each field, neurites were traced and measured using the 2009 ZEN software (Zeiss), and at least 100 cells from three independent experiments were scored for each condition. A cell was considered as neurite-bearing if it contained at least one neuronal process that was longer than the cell body.

### Neuronal primary cultures from rat-brain cerebellum and hippocampus

Primary cultures of cerebellum and hippocampus neurons were prepared from brains of postnatal day (1–2) Sprague Dawley rats as previously described [34,35]. The cerebellum and hippocampus were dissected from the brain and dissociated by papain digestion for 1 h at room temperature, followed by mechanical disaggregation with a Pasteur pipette. Cells were then plated on glass coverslips using 12-well plates at a density of 250,000 cells/well (for confocal microscopy), or on 100-mm culture dishes at a density of 1×10^7^ cells/plate (for subcellular fractionation experiments). Both glass coverslips and culture dishes were pre-coated with 0.01% poly-D-lysine and 10 μg/mL laminin dissolved in PBS. Neuronal cultures were maintained in Neurobasal A media with B27 supplement (Invitrogen), Glutamax, antibiotics (100 U/mL penicillin, and 100 μg/mL streptomycin), and mitotic inhibitors (10 μM uridine + fluoro-deoxyuridine). Cultures were fed every other day by replacing half of the media with fresh, complete media. Neuronal primary cultures were used for confocal microscopy and subcellular fractionation experiments after they became fully differentiated (at least seven days in culture).

### Animal ethics

Experiments using vertebrate animals involved preparation of Primary cultures of cerebellum and hippocampal neurons from brains of postnatal day 1 Sprague Dawley rats. The procedure was done in accordance with the National Institute of Health Guide for the Care and Use of laboratory Animals, and approved by the UTEP Institutional Animal Care and Use Committee (IACUC approval # A-201402-1).

### Differential nuclear staining (DNS) assay for cytotoxicity

To determine the levels of cytotoxicity caused by the experimental compounds (L-28, L-23, PMSF, GRK2i) previously described DNS assay adapted for high-throughput screening was used [36]. This assay uses two fluorescent nucleic acid intercalators, Hoechst 33342 (Hoechst) and propidium iodide (PI). Briefly, PC12 cells were seeded in a 96-well plate format and incubated with NGF and inhibitors. One h before image capturing, cells were added with a staining mixture of Hoechst and PI at a final concentration of 1 μg/mL for each dye. Subsequently, cells were imaged in live-cell mode using a BD Pathway 855 Bioimager system (BD Biosciences, Rockville, MD). Montages (2×2) from four adjacent image fields were captured per well in order to acquire an adequate number of cells for statistical analysis, utilizing a 10× objective. To determine the percentage of dead cells from each individual well, both image acquisition and data analysis were performed using the BD AttoVision v1.6.2 software (BD Biosciences), and each experimental condition was assessed in triplicate.

### Statistical analysis

All statistical analyses were performed using Sigma Plot 11 software (Systat Software, Chicago, IL, USA). In the case of Western blot quantitative analysis, the differences between controls and treatments were assessed by means of the Student’s paired t-test. In the case of neurite outgrowth analysis, the differences in various conditions were assessed by means of one-way ANOVA followed by Holm-Sidak testing (multiple comparisons vs. control). For comparisons between two groups, the Student’s paired t-test was employed, and in all cases, a value of *p <* 0.05 was considered to be statistically significant.

## Results

### NGF-induced neuronal differentiation promotes the interaction of Gβγ with MTs and stimulates MT assembly

**Figure 1:**
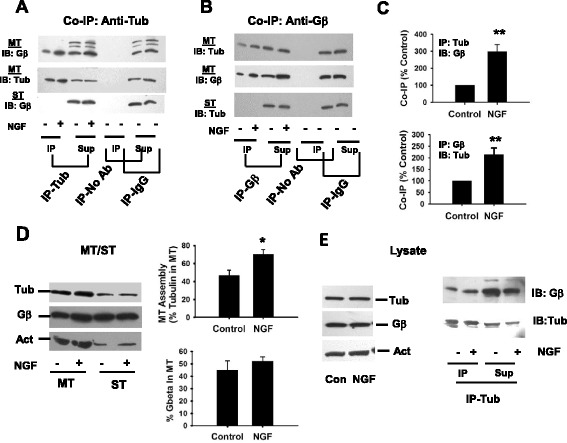
**NGF promotes the interaction of Gβγ with MTs and stimulates MT assembly.** PC12 cells were treated with 100 ng/mL of NGF for three consecutive days. Microtubules (MTs) and soluble tubulin (ST) fractions **(A–D)**, or cell lysates **(E)** were prepared as described in the methods. **(A–C)** Equal amounts of proteins from MT or ST fractions were subjected to co-immunoprecipitation (tubulin and Gβγ) using anti-tubulin **(A)** or anti-Gβ **(B)** followed by immunoblot analysis (Gβ and tub) of immunoprecipitates (IP) and supernatants (SUP) as indicated in the figures. Control experiments include immunoprecipitation in the absence of a primary antibody (No Ab) or in the presence of non-specific rabbit or mouse IgG (IgG). Immunoprecipitation of tubulin or Gβ resulted in co-immunoprecipitation (CO-IP) of tubulin and Gβ. Protein bands (IP) were quantitated and expressed as NGF-induced increase in CO-IP **(C)**. Bar graph shows the mean ± standard error from 3–5 (N) independent experiments as indicated **(C)**. **(D)** Polymerized (MT) and free tubulin (ST) contents as well as the association of Gβ in MT/ST fractions were analyzed by immunoblotting (IB) (left panel). Bar graph represents MT assembly (percent of tubulin in MT) or the percent Gβγ in MT fractions (**D**, right panel) from five independent experiments (mean ± standard error). Loading control include re-probing the blots with anti-actin. **(E)** Representative immunoblots show that NGF does not alter tub or Gβ immunoreactivity in cell lysates (left panel). Loading control include actin. The NGF effect on the increase in co-immunoprecipition of tub and Gβγ (using anti-tub antibody) is shown in the right panel. *p < 0.05; ***p < 0.001.

Assembly and disassembly of MTs is critical for neurite outgrowth and differentiation. Previously we have shown that Gβγ binds to tubulin and promotes MT assembly *in vitro*, and Gβ immunoreactivity was found exclusively in the MT fraction after assembly in the presence of β1γ2, suggesting a preferential association with MTs rather than soluble tubulin [24]. In PC12 cells, we found that Gβγ interacts with MTs and is involved in regulating MT assembly [26]. Because NGF is known to induce neuronal differentiation, we thought that one of the mechanisms by which NGF induces neuronal differentiation could be via Gβγ-MT interactions and changes in MT assembly. To address this, PC12 cells were treated with NGF over the course of three days to allow for neuronal differentiation. Microtubules (MTs) and soluble tubulin (ST) fractions were extracted using a microtubule-stabilizing buffer (MS) as indicated in the methods. The interaction of Gβγ with MT and ST fractions were analyzed by co-immunoprecipitating tubulin-Gβγ complex using a Gβ-specific antibody (rabbit polyclonal anti-Gβ) (Figure 1B and C) or a mouse monoclonal anti-α tubulin antibody (Figure 1A and C), and by determining tubulin or Gβ immunoreactivity respectively in immunoprecipitated (IP) samples. We found that both anti-tubulin and anti-Gβ antibodies could co-immunoprecipitate tubulin-Gβγ complex (Figure 1A and B), and Gβγ was bound preferentially to MTs rather than to dimeric tubulin (ST), which is consistent with our previous studies [24-26]. As predicted, the interaction of Gβγ with MTs was increased significantly (2–3 fold) in NGF-treated cells (Figure 1C). Both Gβ (Figure 1B) and tubulin (Figure 1A) were also immunoprecipitated with respective antibodies. We found that the level of protein immunoprecipitated (tubulin or Gβ) increased to some degree in the presence of NGF although the levels did not correlate with co-immunoprecipated proteins. When immunoprecipitation was performed (control PC12 cells) in the absence of primary antibody (“No ab”) or non-specific rabbit IgG (“IgG”), tubulin- or Gβ- immunoreactivity was not detected in the immunocomplex (Figure 1A and B). This validates the co-immunoprecipitation analysis we have developed to examine tubulin-Gβγ interactions. The result also confirms that the immunoprecipitation experiment can be performed reliably using the MT fraction employed in our study. The MT assembly was assessed by determining tubulin immunoreactivity in MT and ST fractions and measuring the ratio of tubulin incorporated in the MTs vs. free tubulin as a direct measure of MT assembly (Figure 1D). We found that MT assembly was stimulated significantly (from 45.3 ± 4.8% to 70.1 ± 3.6%) in NGF-differentiated PC12 cells (Figure 1D). Loading control includes re-probing the blots with anti-actin. To determine whether protein expression was affected after NGF treatment, cell lysates were prepared and subjected to western blotting. Representative immunoblots show that NGF does not alter tubulin or Gβ immunoreactivity in cell lysate (Figure 1E, left panel). The effect of NGF on the increase in co-immunoprecipition of tubulin and Gβγ (using anti-tub antibody) is shown in the right panel. Previously, using the anti-microtubule drug nocodazole, we have shown that the interaction of Gβγ with MTs is an important determinant for MT assembly. While microtubule depolymerization by nocodazole inhibited the interactions between MTs and Gβγ, this inhibition was reversed when microtubule assembly was restored by the removal of nocodazole [26].

Although it can be argued that MT structure is no longer intact in MT fraction subsequent to sonication and low-speed centrifugation, we have shown earlier that the tubulin dimer binds to Gβγ and that the tubulin-Gβγ complex preferentially associates with MTs [24,25]. Therefore, tubulin-Gβγ complex is expected to be present in the MT fraction prepared in this study. The absence of any interaction between Gβγ and tubulin in the ST fraction in spite of their presence further supports this result (Figure 1A). Furthermore, tubulin oligomers are expected to be present in the MT fraction, and the possibility exists that Gβγ preferentially binds the oligomeric structures [24]. The increased interactions of Gβγ with MTs and the stimulation of MT assembly observed in the presence of NGF could allow for a rearrangement of MTs during neuronal differentiation.

**Figure 2:**
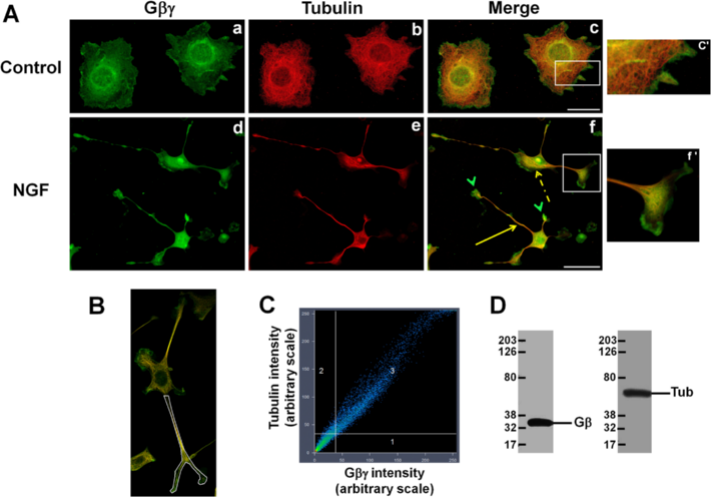
**Gβγ co-localizes with MTs in the neuronal processes in NGF-differentiated PC12 cells.** PC12 cells were treated with and without NGF (control). **(A)** The cells were then fixed and double labeled with anti-tubulin (red) and anti-Gβ (green) antibodies as indicated in the methods. Areas of overlay appear yellow. The enlarged image of the white box **(c)** shows co-localization of Gβγ with MTs in the perinuclear region **(c’)**. The white box on the lower panel **(f’)** shows the enlarged growth cone, with Gβγ co-localizing with tubulin along the neuronal process and in the central portion of the growth cone, while the neuronal tips show predominant Gβγ immunostaining. The solid yellow arrow indicates neuronal processes, and the broken yellow arrow indicates cell body. Green arrowhead indicates only Gβ labeling (not tubulin) at the neuronal tips. The scale bars in **“a–c”** and **“d–f”** are 20 μm and 50 μm, respectively. **(B)** Co-localization of Gβγ with MTs in the neuronal processes was quantitatively assessed using Zeiss ZEN software. A representative image of a region of interest (neuronal process) of an NGF-differentiated PC12 cell is shown. **(C)** A representative scattergram depicting co-localization of Gβγ with MTs along the neuronal process is shown. **(D)** Representative Western blots (using PC12 whole-cell lysates) showing the specificity of the anti-Gβ (left) and anti-tubulin (right) antibodies that were used for immunofluorescence.

The interaction of Gβγ with MTs in NGF-differentiated cells was also assessed by immunofluorescence microscopy. PC12 cells that were treated with and without NGF were examined for Gβ and tubulin by confocal microscopy. Tubulin was detected with a monoclonal anti-tubulin (primary antibody) followed by a secondary antibody (goat-anti-mouse) that was labeled with tetramethyl rhodamine (TMR). Similarly, Gβγ was identified with rabbit polyclonal anti-Gβ followed by FITC-conjugated secondary antibody (goat-anti-rabbit), and the cellular localizations and co-localizations were recorded by laser-scanning confocal microscopy. In control cells (in the absence of NGF), Gβγ co-localized with MTs in the cell body as well as the perinuclear region (Figure 2A, a–c; see also enlargement in c’). After NGF treatment, the majority of the cells displayed neurite formation (Figure 2A, d–f). Gβγ was detected in the neurites (solid arrow, yellow) and in cell bodies (broken arrow, yellow), where they co-localized with MTs. Interestingly, Gβγ was also localized at the tips of the growth cones (Figure 2A, f), where very little tubulin immunoreactivity was observed (green arrowhead). The enlarged image of the white box in f (Figure 2A, f’) indicates the co-localization of Gβγ with MTs/tubulin along the neuronal process and in the central portion of the growth cone, but not at the tip of the growth cones. To quantitatively assess the overall degree of co-localization between Gβγ and MTs/tubulin along the neuronal processes, an entire neuronal process was delineated as a region of interest (ROI) using a white contour (Figure 2B), and the co-localization scattergram (using Zeiss ZEN 2009 software) is shown in Figure 2C, in which green (Gβγ) and red (tubulin) signals were assigned to the *x* and *y* axes, respectively. Each pixel is presented as a dot, and pixels with well co-localized signals appear as a scatter diagonal line. The average Manders’ overlap coefficient (0.91 ± 0.014) suggests a robust co-localization between Gβγ and tubulin along the neuronal process. We found that ~60% of cells exhibit strong co-localization between Gβγ and tubulin (Manders’ overlap coefficients 0.9 or above) in the presence of NGF. Rest of the cells also showed high degree of co-localization ranged from 0.6 to 0.87. The specificities of the antibodies are demonstrated in Figure 2D, in which the monoclonal anti-α tubulin antibody appears to be highly specific for tubulin in PC12 cells and the polyclonal anti-Gβ antibody we used for the immunofluorescence studies does not show any cross reactivity with other proteins in PC12 cells.

### Gβγ-binding peptides affect MT organization, cellular morphology, and neurite formation in NGF- differentiated PC12 cells

To better understand the role of Gβγ in MT organization and neurite outgrowth, we used two synthetic Gβγ-binding peptides GRK2i, and mSIRK. GRK2i, a Gβγ-inhibitory peptide, corresponds to the Gβγ-binding domain of GRK2 (G-protein-coupled receptor kinase 2) and selectively prevents Gβγ-mediated signaling and has therefore been a valuable tool for understanding Gβγ-dependent functions in cell culture systems [37-41]. On the other hand, mSIRK is known to activate Gβγ signaling in cells by promoting the dissociation of Gβγ from α subunits without a nucleotide exchange [42,43].

**Figure 3:**
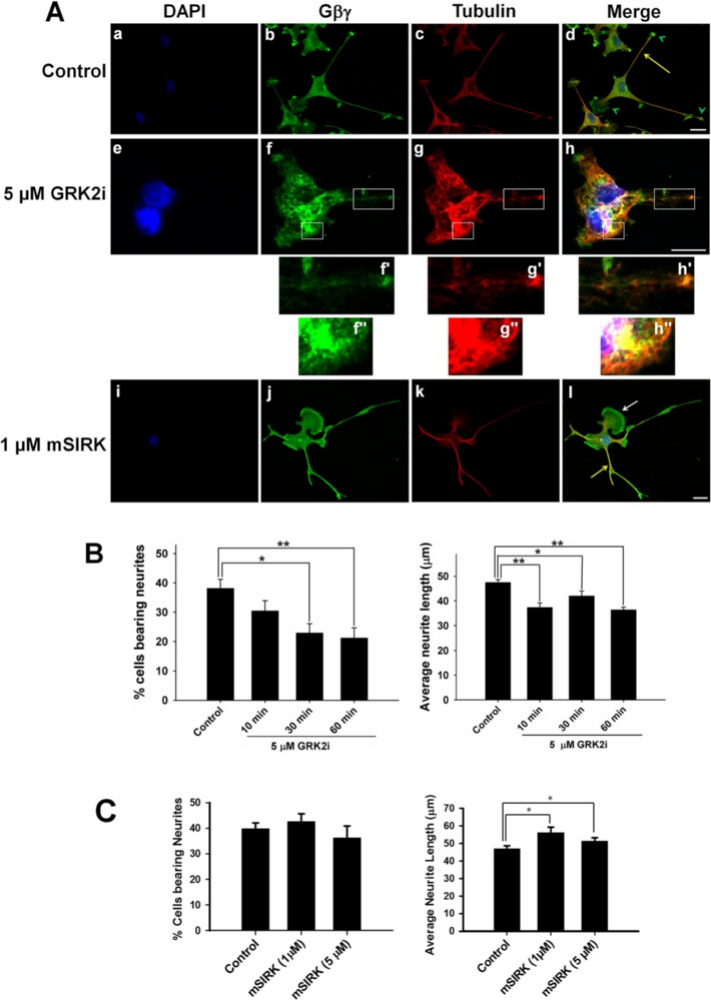
**Effect of Gβγ-binding peptides, GRK2i, and mSIRK on MTs and Gβγ organization and neurite outgrowth. (A)** PC12 cells were treated with 100 ng/mL of NGF for two consecutive days. Subsequently, 5 μM GRK2i was added to the media and the cells were incubated for 10, 30, and 60 min as indicated. The cells were then fixed and double labeled with anti-tubulin (red) and anti-Gβ (green) antibodies and processed for confocal microscopy. DAPI was used for nuclear staining (blue). Control cells exhibit typical neuronal morphology, displaying long neurites. Gβγ is shown to co-localize with tubulin/MTs along the neuronal processes (solid yellow arrow) but not at the tip of the neurites (green arrowheads), where Gβγ immunostaining is predominant. Inhibition of Gβγ signaling by incubation with GRK2i causes neurite damage, microtubule disruption, and alters the Gβγ-tubulin co-localization pattern as shown in the enlarged images in the white boxes (**f’–h’**, and **f”–h”**). To test the effect of mSIRK, PC12 cells were treated with mSIRK (1 μM) for 2 h, followed by 1-day treatment with NGF. Scale bars are 20 μm. **(B–C)** PC12 cells were treated with GRK2i or mSIRK as described above, followed by fixing and processing for confocal microscopy. Using Zeiss ZEN software, neurites were traced and measured, and average neurite length and percent of cells bearing neurites were determined. Differences between experimental conditions were assessed by one-way ANOVA. *p < 0.05; **p < 0.01.

To test the effect of GRK2i, PC12 cells were treated with 100 ng/mL of NGF for two consecutive days to induce neurite outgrowth. Subsequently, 5 μM GRK2i was added to the media and the cells were incubated for 10, 30, and 60 min as indicated in the figure (Figure 3). The cells were then fixed and double labeled with anti-tubulin (red) and anti-Gβ (green) antibodies, and processed for confocal microscopy. DAPI was used for nuclear staining (blue). Control cells exhibit typical neuronal morphology, displaying long neurites (Figure 3A (a-d). Gβγ is shown to co-localize with tubulin/MTs along the neuronal processes (solid yellow arrow). As indicated in Figure 3A (e–h), neurite damage (enlarged images f’, g’, and h’) as well as MTs and Gβγ aggregation (enlarged images f”, g”, h”) was observed in the presence of 5 μM GRK2i. In addition, cellular aggregation was also frequently observed in the presence of GRK2i. Images shown here were taken after 60 min of incubation with GRK2i. We used higher magnification and enlarged images of GRK2i-treated cells to show neurite damage, MT disruption, and cellular aggregation. Measurement of the number and length of neurites provides a quantitative assessment of neuronal differentiation [44]. Therefore, the effect of GRK2i on neuronal differentiation was assessed by measuring average neurite lengths as well as the percentage of cells bearing neurites (Figure 3B) as described in the methods. A cell was considered neurite-bearing if it contained at least one neuronal process that was longer than the cell body (13.7 ± 0.5 μm in diameter). As indicated in Figure 3B and C, the percentage of cells bearing neurites was reduced significantly—from 38.1 ± 3.1% in control cells to 22.8 ± 3.1% after 30 min of incubation with GRK2i—and did not reduce further after 60 min of incubation. The average neurite length of surviving neurites decreased modestly in the presence of GRK2i and increasing the incubation time from 10 min to 60 min did not have any additional effect. To better understand the role of GRK2i, we pre-incubated PC12 cells with GRK2i for 2 h and allowed them to differentiate in the presence of NGF. We found that the effect of GRK2i on the average neurite length, as well as on the cells bearing neurites, were quite similar to that observed with the post-incubation of preformed neurites with GRK2i (Additional file 1: Figure S1).

We found that mSIRK (1 and 5 μM) did not inhibit neurite outgrowth but rather increased average neurite length (Figure 3C). Interestingly, many of the neurites formed in the presence of mSIRK were longer compared with control cells and had morphology similar to that observed in Gβγ overexpressed cells, which could be due to the fact that mSIRK can increase the free Gβγ pool in a cell similar to Gβγ overexpression. This observation is supported by a recent report by Garcia-Oliveres et al. [43] in which the authors found that Gβγ overexpression, or treatment with the Gβγ activator mSIRK, resulted in rapid inhibition of dopamine-transporter (DAT) activity in cells.

### Inhibitors of prenylated methylated protein methyl esterase (PMPMEase) disrupt MTs and Gβγ organization and affect neurite formation

**Figure 4:**
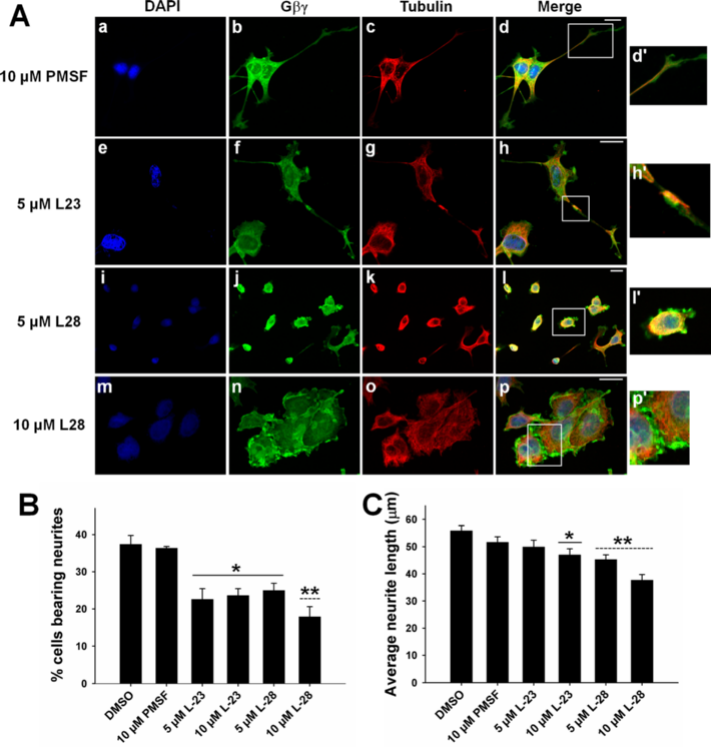
**Inhibitors of PMPMEase disrupt neurite outgrowth of NGF-differentiated PC12 cells.** PC12 cells were treated with PMPMEase inhibitors, L-23 and L-28 (5 μM, and 10 μM), or the prototypical molecule PMSF (10 μM) and allowed to differentiate in the presence of 100 ng/mL of NGF for two consecutive days. **(A)** The cells were then fixed and double labeled with anti-tubulin (red) and anti-Gβ (green) antibodies, and DAPI was used for nuclear staining (blue). Co-localization patterns are also shown in the merged images. PMSF did not seem to have any significant effects on neuronal morphology **(a–d)**. PMPMEase inhibitors inhibited neurite outgrowth of NGF-treated PC12 cells, causing axonal damage (**e–h**, enlarged image shown in **h’**), neurite shortening (**i–l**, enlarged image shown in **l’**), and cellular aggregation **(m–p)**. Scale bars are 20 μm **(B–C)**. Using Zeiss ZEN software, neurites were traced and measured, and the average neurite length and percent of cells bearing neurites were estimated. The differences between experimental conditions were assessed by one-way ANOVA. *p < 0.05; **p < 0.01.

A number of proteins, including the γ subunit of Gβγ, undergo a process of post-translational modification termed prenylation, and this modification is important for the biological functions of these proteins. Earlier, we have shown that prenylation of the γ subunit of Gβγ is important for the interaction of Gβγ with tubulin and stimulation of MT assembly *in vitro* [24,25]. The prenylation pathway consists of three enzymatic steps, the first of which is the addition of a prenyl group to the cysteine residue of the carboxy-terminal CAAX motif, followed by the cleavage of the tripeptide (AAX). The terminal carboxylic acid group then undergoes methylation, which is catalyzed by the prenylated protein methyl transferase (PPMTase, also known as isoprenylcysteine carboxylmethyltransferase or ICMT). PMPMEase readily hydrolyzes ester bonds of the methylated prenylated proteins, thus making the methylation step reversible [45-47]. Using phenylmethylsulfonyl fluoride (PMSF) as a prototypical molecule, Aguilar et al. [27] have recently synthesized high-affinity-specific inhibitors of PMPMEase and two such inhibitors (L-23 and L-28) have been shown to induce degeneration of human SHSY5Y neuroblastoma cells, suggesting that this enzyme plays a possible role in neuronal survival [27,45]. Therefore, we used L-23 and L-28 (Figure 4) in this study to determine whether PMPMEase may play a regulatory role in the Gβγ-dependent regulation of MTs and neurite outgrowth. Two separate approaches were used to test the effect of the inhibitors. First, PC12 cells were treated with the PMPMEase inhibitors (L-23, L-28), or PMSF (1, 5, or 10 μM) overnight, and then allowed to differentiate in the presence of NGF for two consecutive days. Second, PC12 cells were treated with 100 ng/mL of NGF over the course of two days, followed by overnight treatment with L-28, L-23, or the prototypical molecule PMSF. Both approaches essentially produced a similar effect. At 1-μM concentration, the inhibitors did not have any noticeable effect on neurite outgrowth (figure not shown). As shown in the figure (Figure 4), pretreatment with both inhibitors significantly affected NGF-induced neurite outgrowth, with L-28 being more potent. Confocal microscopic examination shows neurite damage (Figure 4A, e–h; see the enlarged image in the box), inhibition of neurite outgrowth (Figure 4A, i–l), and altered organization of the MTs and Gβγ. Cellular aggregation was also evident in the presence of 10 μM L-23 or L-28. Again, the effect was more potent in the presence of L-28 (Figure 4A, m–p). As indicated in Figure 4A (m–p), Gβγ was concentrated in the cell-cell contact region (clearly visible in the enlarged box) in the presence of 10 μM L-28 and could be responsible for mediating cellular aggregation. The effects of L-23 and L-28 on neuronal outgrowth were assessed quantitatively by measuring average neurite lengths as well as the percentage of cells bearing neurites as was done previously in the presence of GRK2i. As indicated in Figure 4B and C, the percentage of cells bearing neurites was reduced significantly in the presence of 5 or 10 μM L-23 and L-28, with L-28 at 10 μM being the most potent. The average neurite length of surviving neurites was also decreased modestly in the presence of 10 μM L-23, or 5 μM and 10 μM L-28. Once again, L-28 at 10 μM appeared to be the most potent in inhibiting neurite outgrowth. The effect of PMPMEase inhibitors in preformed neurites (post-treatment with L-23 and L-28) is shown in Additional file 2. As shown in the figure (Additional file 2), the effect of inhibitors is essentially similar to that observed in Figure 4, except that average neurite lengths were unaffected by L-23. We also tested the effect of PMPMEase inhibitors in PC12 cells in the absence of NGF to determine whether the MT cytoskeleton is affected in undifferentiated PC12 cells (Additional file 3). As shown in the figure (Additional file 3) disruption of MTs, altered cellular localization of Gβγ, as well as cellular aggregation was also observed in control PC12 cells. The result further suggests that neurite damage observed in the presence of PMPMEase inhibitors might be due to the disruption of Gβγ-MT mediated pathways.

**Figure 5:**
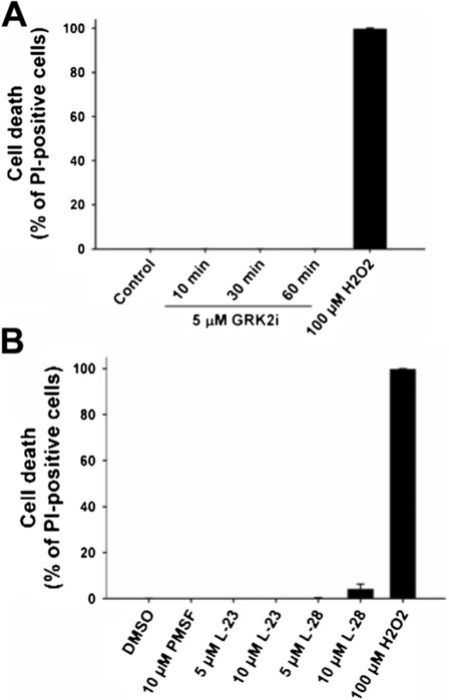
**Inhibitors of PMPMEase and GRK2i do not induce neuronal cell death.** PC12 cells were grown on 96-well plates and treated with NGF for two days followed by incubation with 5 μM GRK2i for 10, 30, and 60 min **(A)**. For PMPMEase inhibitors treatment, cells were seeded on 96-well plates and incubated simultaneously with NGF and PMSF, L-23, or L28 (5 and 10 μM) for two days **(B)**. Subsequently, cells were incubated with a Hoechst/propidium iodide (PI) mixture for DNS cytotoxicity assay. The images were captured in live-cell-image mode using the confocal automated microscope BD Pathway Bioimager System and a 10× objective, assisted with AttoVision software. H_2_O_2_ (100 μM) was used as a positive control. Cell nuclei stained with Hoechst provided the total number of cells; cell nuclei stained with PI indicate the number of dead cells; merged Hoechst and PI images. Cell death was plotted as the percent of PI-positive cells, denoting the total number of dead cells for each condition.

Since neurodegeneration occurs in the presence of Gβγ-inhibitory peptide GRK2i or PMPMEase inhibitos L-23 and L-28, it is necessary to demonstrate that the inhibitors are not toxic to the cells under the experimental conditions used for this study. To determine the levels of cytotoxicity caused by L-28, L-23, or GRK2i, previously described DNS assay adapted for high-throughput screening was used [36]. This assay uses two fluorescent nucleic acid intercalators, Hoechst 33342 (Hoechst) and propidium iodide (PI). Hoechst has the ability to cross cell membranes of both healthy and dead cells and to stain nuclear DNA, thus providing the total number of cells, whereas PI is only able to stain cells having a loss of plasma-membrane integrity, thus denoting the number of dead cells. In the case of GRK2i treatment, PC12 cells were grown on 96-well plates and induced to differentiate in the presence of NGF for two days, followed by incubation with 5 μM GRK2i for 10, 30, and 60 min. For PMPMEase inhibitors treatment, cells were seeded on 96-well plates and incubated simultaneously with NGF and PMSF, L-23, or L28 (5 and 10 μM) for two days. Cells were then incubated with a mixture of Hoechst/propidium iodide (PI). Subsequently, cells were imaged in live mode using a BD Pathway 855 Bioimager system as described in the methods section. The percentage of dead cells in the presence of inhibitors was determined by using the BD AttoVision v1.6.2 software (BD Biosciences) and the result was plotted as shown in the figure (Figure 5). As indicated in the figure, GRK2i did not cause cytotoxicity on NGF-differentiated PC12 cells. In the case of the PMPMEase inhibitors L-23, no cell death was detected at the tested concentrations. Cell death starts to appear at 10 μM L-28, and could account for cellular aggregation observed in the presence of 10 μM L-28 (Figure 4, m-p). The prototypical compound, PMSF, was also assayed and not found to be cytotoxic. Hydrogen peroxide (100 μM) was used as a positive control.

### Overexpression of Gβγ in PC12 cells induces neurite outgrowth: Overexpressed Gβγ co-localizes with MTs in the neuronal processes

**Figure 6:**
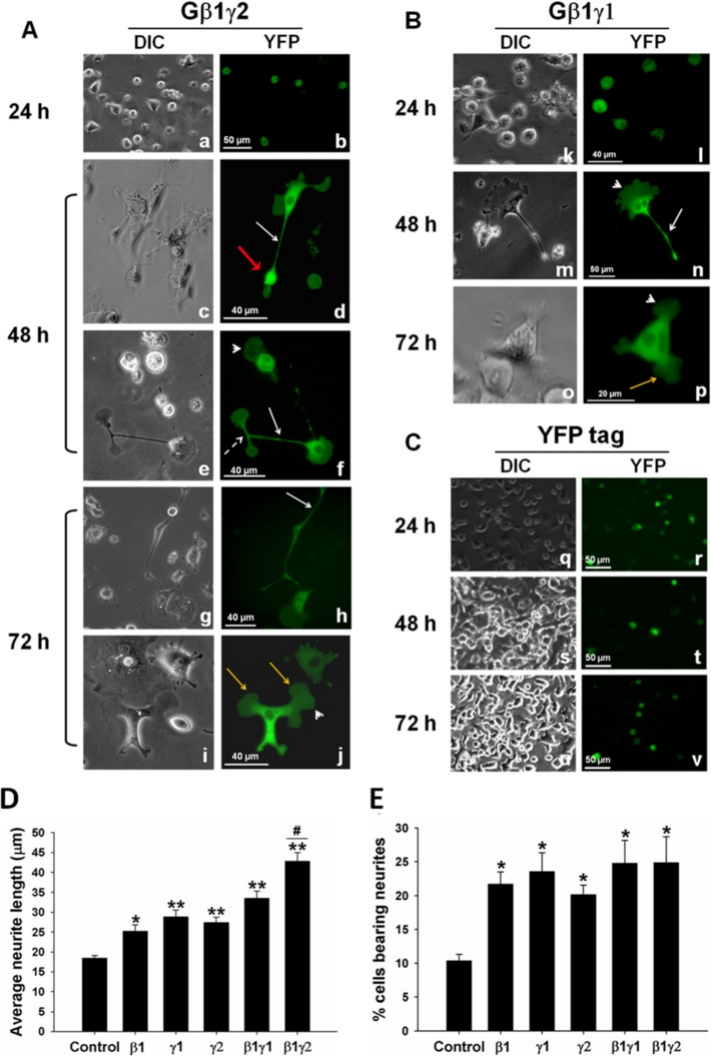
**Overexpression of Gβγ induces neurite outgrowth in PC12 cells.** PC12 cells were co-transfected with YFP-tagged constructs encoding **(A)** Gβ1 and Gγ2 (β1γ2) or with **(B)** Gβ1 and Gγ1 (β1γ1) in the absence of NGF, using Lipofectamine LTX PLUS reagent according to manufacturer instructions. Cells overexpressing fluorescent proteins were monitored at different time points (24, 48, and 72 h) for protein expression and morphological changes using a fluorescence microscope. Images taken with DIC and YFP filters are shown. **(C)** PC12 cells transfected with a plasmid-encoding YFP only was used as control and observed through the same time points. Neuronal processes, white arrows; growth cones, red arrows; axonal branching, broken white arrow; cytoskeletal labeling, white arrowhead; enlarged and bulky neurites, yellow arrows. **(D and E)** Neurites were traced and measured using the 2009 ZEN software from Zeiss. At least 100 cells from three independent experiments were measured for each preparation, and average neurite length and percent of cells bearing neurites calculations and statistical analysis were done using SigmaPlot software. **(D)** The average neurite length of Gβ1-, Gγ1-, Gγ2-, Gβ1γ1- and Gβ1γ2- overexpressing PC12 cells. **(E)** The percentage of cells bearing neurites in transfected cells was also estimated. *p value < 0.05; **p value < 0.005 when compared to control. ^*#*^ *p* value = 0.005 when compared with β1γ1.

To further elucidate the role of Gβγ in neuronal differentiation, we overexpressed Gβγ in PC12 cells. Since previous studies have indicated that Gβ1γ2 promoted MT assembly *in vitro*—and Gβ1γ1 was without any effect [24]—PC12 cells were transfected with either β1γ1 or β1γ2. YFP-tagged β1, γ2, or γ1 constructs were used for transfection. Cells were co-transfected with β1 and γ2, β1 and γ1, or individual constructs (Gβ1, Gγ1, and Gγ2). A plasmid encoding only YFP was used as control. Cells were monitored for protein expression and for possible neurite formation at different time points (24, 48, and 72 h). Both DIC and fluorescent images of the live cells are shown in Figure 6.

We found that within 24 hours of transfection, both β1γ1 and β1γ2 transfected PC12 cells were found to overexpress the proteins as demonstrated by the fluorescent (YFP) labeling. DIC images indicated no changes in morphology (Figure 6A, a–b; 6B, k–l). At 48 h of transfection, YFP-β1γ2 transfected cells induced neurite formation (in the absence of NGF). Overexpressed protein (YFP-Gβ1γ2) was localized in the neurite processes (white arrows), growth cones (red arrows), and cell bodies as shown by fluorescent (YFP) labeling (Figure 6A). Higher magnification was used (Figure 6, c-j, m-p) to show the details of the morphological changes observed in Gβγ-overexpressed PC12 cells. For example, Cytoskeletal labeling (Figure 6f, arrowhead) was observed in higher magnification in some cells, suggesting the localization of the protein with cytoskeletal filaments. Interestingly, we found that many of the β1γ2 overexpressed cells had a tendency to divide into two equal halves at the tip of the neurites (dashed arrow). After 72 hours, some cells displayed complex neurite formation (Figure 6A, g-h), but in many cells the neurites became shortened and the tips became enlarged (Figure 6A, i-J; yellow arrows). As indicated in the figure (Figure 6B), Gβ1γ1-transfected PC12 cells also induced neurite formation although to a lesser extent than Gβ1γ2-transfected cells as determined by live microscopy and quatitative analysis of neurite length (Figure 6D and E). Control cells overexpressing only YFP did not induce neurite formation after 48 or 72 h of transfection (Figure 6C). The addition of NGF (100 ng/mL) did not have any additional effect on neurite formation in Gβγ-overexpressed cells. Because both Gβ and Gγ constructs used in the current study were YFP tagged, it was not possible to evaluate whether cells that induced neurites were overexpressed with both subunits or not. However, when PC12 cells were transfected with individual constructs (Gβ1, Gγ1, and Gγ2), they all induced neurites (live images are not shown), although average neurite lengths were less than that observed in the presence of Gβ1γ2 or Gβ1γ1 (Figure 6D and E).

To assess neurite outgrowth in Gβγ-overexpressing cells, average neurite lengths as well as the percentage of cells bearing neurites were measured in Gβ1-, Gγ1-, Gγ2-, Gβ1γ1-or Gβ1γ2-overexpressed cells (Figure 6D and E). Overexpressed cells (48 h) were fixed and processed for confocal microscopy using a mouse monoclonal anti-tubulin antibody, followed by labeling with rhodamine (TMR) conjugated secondary antibody. The overexpressed cells (YFP-tagged) were only imaged using rhodamine staining for the purpose of neurite outgrowth assessment. Cells were viewed using the 40× objective with a Zeiss LSM 700 confocal microscope. The coverslips were scanned from left to right, and 8–10 fields were randomly selected. For each field, neurites were traced and measured using the 2009 ZEN software (Zeiss) and at least 100 cells from three independent experiments were scored for each condition. A cell was considered neurite bearing if it contained at least one neuronal process that was longer than the cell body (15.59 ± 0.5 μm in diameter). The average neurite length of Gβ1γ2 (42.8 ± 2.1 μm) and Gβ1γ1 (33.5 ± 1.8 μm) is significantly higher than that of control cells (18.4 ± 0.6 μm), with Gβ1γ2 having the most potent effect on neurite outgrowth. Cells overexpressing singly with Gβ or Gγ subunits also exhibited an increase in average neurite lengths compared to control cells as indicated in the figure (Figure 6D and E). Although the average neurite length in Gβγ-overexpressing cells (42.8 ± 2.1 μm) was slightly lower than that observed in NGF-differentiated PC12 cells (53.6 ± 1.8 μm), the result clearly indicates the effectiveness of Gβγ in inducing neurite outgrowth. We also evaluated the percentage of cells bearing at least one neurite in cells in each condition. We found that ~25% of the Gβ1γ2-overexpressing cells induced at least one neurite (Figure 6E). About 10% of control cells overexpressing only YFP induced short neurites was also observed in PC12 cells in the absence of NGF.

**Figure 7:**
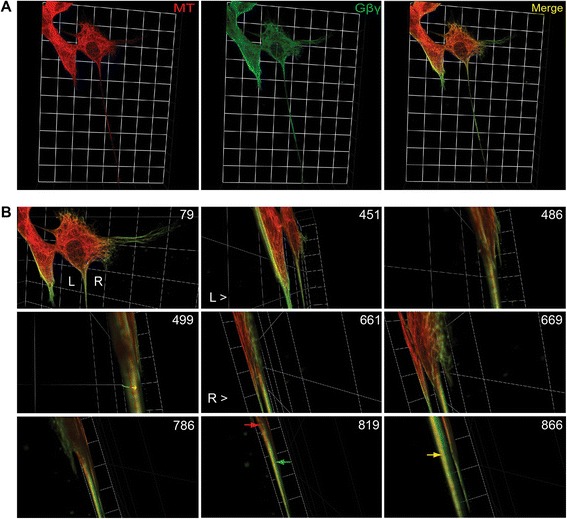
**Three-dimensional (3-D) view of co-localization of Gβγ and microtubules (MTs).** Co-localization of overexpressed Gβγ (green) with MTs (red) as visualized by high-resolution 3-D confocal images using Volocity software (see Methods). The images shown in this assembly are still frames from Additional file 4: Movie 1 (Supplementary materials). **(A)** A still frame from the movie separated into its component channels: MT (red) and Gβγ (green) expression are each confined discretely to similar subcellular locations as shown in the merged panel (yellow). **(B)** Representative still frames were selected to summarize the movie content. The numbers on the top right of each still image denote the frame numbers within the movie. Arrows in frame 819 correspond to MT expression (red, top arrow) and Gβγ (green, bottom arrow) expression. The arrow in frame 866 points to co-localization of MT and Gβγ (yellow). The edges of each individual square in the background grid for each image are 19.21 μm in length. For detailed description, please see the text.

To test the localization and association of overexpressed Gβγ (YFP-Gβ1γ2) with MTs, cells overexpressing Gβγ (48 h) were fixed and processed for confocal microscopy (Figure 7) as previously done with NGF-differentiated cells. Tubulin was detected with a monoclonal mouse anti-tubulin antibody followed by a secondary antibody (goat anti-mouse) that was labeled with tetramethyl rhodamine. Gβγ and MTs were visualized with high-resolution 3-D reconstructions of confocal image stacks using Volocity 3-D Image Analysis Software. Rotations performed on the deconvolved 3-D reconstruction within the software’s graphical user interface allowed the transfected PC12 cells to be viewed from any direction for a more complete picture of the neuronal processes. The localization of Gβγ in neuronal processes and its association with MTs were clearly visible by panning, zooming, and rotating the 3-D images. Bookmarking the time points at which we performed these translations of the reconstruction allowed for capture within a motion picture format (see Additional file 4) and the extraction of still frames (Figure 7). MT filaments (red; Figure 7A, left panel, and Figure 7B, Frame 819) and Gβγ (green; Figure 7A, middle panel, and Figure 7B, Frame 819) interact throughout the neuronal process as evidenced by clear yellow labeling (Figure 7B, Frame 866). Gβγ labeling (green) was also observed from all directions to be alongside yellow labeling throughout the neuronal process (Figure 7B, Frames 499, 669, 786, 819, and 866). In some areas, red labeling was also clearly visible. The labeling pattern appears to support our *in-vitro* results, which indicate that Gβγ binds on the microtubule wall when promoting MT assembly [24]. These results are also consistent with the possibility that the yellow labeling we observe in neurites marks domains on Gβγ that interact with MT filaments, and that the green labeling represents Gβγ domains that are not interacting directly with MTs but projecting from MT walls. These possibilities notwithstanding, it is reasonable to suggest on the basis of this unique labeling pattern as well as on previous *in-vitro* results [24] that Gβγ induces neurite outgrowth through its ability to interact with tubulin/MTs and stimulate MT assembly.

### Gβγ interacts with MTs in hippocampal and cerebellar neurons cultured from rat brains

**Figure 8:**
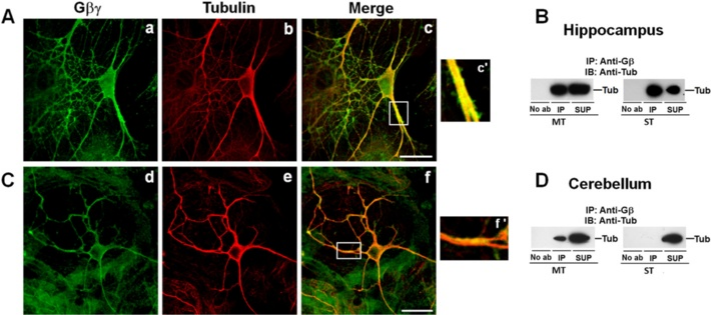
**Gβγ interacts with MTs in primary hippocampal and cerebellar neurons.** Neuronal primary cultures from hippocampus **(A, B)** and cerebellum **(C, D)** of rat brains were prepared as described in the methods. Hippocampal **(A)** and cerebellar **(C)** neurons were processed for confocal microscopy using anti-tubulin (red) and anti-Gβ (green) antibodies. Areas of overlay appear yellow. The enlarged view of the white boxes **(c’, f’)** depicts Gβγ-tubulin co-localization in the neuronal process in hippocampal and cerebellar neurons. The scale bar is 20 μm. Microtubules (MT) and soluble tubulin (ST) fractions were prepared from hippocampal **(B)** and cerebellar **(D)** neurons as described in the methods. Equal amount of proteins from each fraction were subjected to co-immunoprecipitation using anti-Gβ antibody or in the absence of primary antibody (No ab) followed by an immunoblot analysis of immunoprecipitates (IP) and supernatants (SUP) using anti-α-tubulin antibody **(B, D)**.

Although PC12 cells have been used extensively to study the mechanism of neuronal outgrowth and differentiation, neurons are more complex and give rise to a “dendritic tree” and an axon that may branch hundreds of times before it terminates. The axon terminal contains synapses—specialized structures that release neurotransmitters in order to communicate with target neurons. Thus, neurons are capable of interacting to form the complex neuronal networks necessary for the processing and transmission of cellular signals. To precisely identify the role of Gβγ-MTs interactions in neuronal morphology and functioning, it is important to demonstrate whether this interaction occurs in neurons. Therefore, as a first step we established neuronal primary cultures from newborn rat brains, specifically from the cerebellum and hippocampus. These brain regions were selected because they have been extensively validated as cell-culture models for studying the role of the cytoskeleton in neuronal polarity and axonal development [48-50]. In addition, these two brain regions are associated with different functions. While the hippocampus is involved in memory formation and neural plasticity, the cerebellum is responsible for motor control, posture, and balance [51,52]. As described with PC12 cells, confocal microscopy, subcellular fractionation, and co-immunoprecipitation analysis were performed to determine the co-localization/interactions of Gβγ with MTs in hippocampal and cerebellar neurons. We found that Gβγ co-localizes very intensely with MTs in the neuronal processes in hippocampal neurons (Figure 8A, panels c and c’). Co-immunoprecipitation analysis using MT and ST fractions indicates that Gβγ interacts with both MTs and STs in hippocampal neurons (Figure 8B). In cerebellar neurons, both confocal microscopy (Figure 8C) and co-immunoprecipitation analyses (Figure 8D) indicate a weak association of Gβγ with MTs.

## Discussion

The results presented here demonstrate that the regulated interaction of Gβγ with MTs may be critical for neurite outgrowth and differentiation, and that NGF could facilitate the process by promoting this interaction. In addition, prenylated methylated protein methyl esterase (PMPMEase) appears to be a critical regulator of this interaction. This conclusion is supported by four main lines of evidence: (1) NGF-induced neurite outgrowth promotes the interaction of Gβγ with MTs and stimulates MT assembly, (2) Gβγ − binding peptides affect MT organization and neurite formation, (3) inhibitors of PMPMEase (an enzyme involved in the prenylation pathway) disrupts Gβγ and MT organization and neurite outgrowth, and (4) overexpression of Gβγ induces neurite outgrowth in the absence of NGF.

Although Gβγ has been shown to bind to tubulin and promote MT assembly *in vitro* and in PC12 cells [24-26,53], the functional implication of this interaction has not been demonstrated. Reports from several laboratories have indicated the involvement of Gβγ in neuronal development and differentiation [17,54], and recently Gβ1-deficient mice have been shown to have neural-tube defects [55]. Earlier, it was shown that impaired Gβγ signaling promoted neurogenesis in the developing neocortex and increased neuronal differentiation of progenitor cells [54]. Our data suggest that the interaction of Gβγ with MTs and its ability to stimulate MT assembly may provide a mechanism by which Gβγ regulates neuronal differentiation. Based on our high-resolution image analysis of the neuronal processes induced by overexpression of Gβγ (Figure 7), it appears that MT filaments and Gβγ interact throughout the neuronal processes. Gβγ labeling was also observed side by side with MT labeling from all directions. This labeling pattern appears to support our earlier *in-vitro* results, which indicate that Gβγ binds on the microtubule wall [24]. The observed interaction of Gβγ with MTs in hippocampal and cerebellar neurons (Figure 8) further supports the role of Gβγ-MT interaction in neuronal development and differentiation.

It was observed that overexpression of Gβ1γ1 also induced neurite formation although to a lesser extent than Gβ1γ2-overexpressed cells as observed by live microscopy and quantitative analysis of neurite length (Figure 6B-D). Using purified proteins (*in vitro*) we had previously demonstrated earlier that only β1γ2 but not β1γ1 binds to tubulin with high affinity and stimulates MT assembly [24,25]. However, *in vivo*, overexpressed β1 or γ1 may interact with endogenous β or γ subtypes to some degree to form various βγ combinations including β1γ2, which could be responsible for the observed effect of β1γ1 overexpression (neurite formation) in PC12 cells. Furthermore, it is likely that the weaker affinity of Gβ1γ1 with tubulin observed *in vitro* using purified proteins [24,25] became amplified in the presence of other cellular component(s) *in vivo*. Nonetheless, the results clearly demonstrate that the Gβ1γ2 is more potent in inducing neurite outgrowth compared to Gβ1γ1.

Previously we have shown that prenylation and further carboxy terminal processing (methylation) of the γ subunit of Gβγ are important for interaction with MTs and stimulation of MT assembly *in vitro* [24]. We decided to target the post-prenylation processing enzyme PMPMEase in this study for two reasons. First, although prenylation has been studied extensively because of the prevalence of prenylated proteins in cancer biology—and the prenyl transferase enzyme has been targeted for clinical trials—the results so far have not been promising; therefore, attention has recently been diverted to post-prenylation pathways. The enzyme involved in methylation of the prenylated protein, isoprenylcysteine carboxyl methyltransferase (ICMT), is now being studied for cancer metastasis and results appear to be promising [56]. More recent studies have indicated that targeting ICMT might be useful in treating the rare genetic disease progeria [57]. Second, inhibitors for PMPMEase have recently been synthesized and shown to induce degeneration of human neuroblastoma SHSY5Y cells [27]. Although the γ subunit of Gβγ may not be the only target of PMPMEase (the Rho and Ras families of GTPases also undergo prenylation and subsequent methylation/demethylation), based on previous findings, the major protein that undergoes *in*-*vivo* methylation in rat brains in response to injection of endogenous methyl donor S-adenosyl methionine is a molecule with a molecular weight comparable to that of the γ subunit of G proteins [58,59]. Therefore, it is likely that the γ subunit of the G protein was a major target of PMPMEase inhibition in our experiment. We found that NGF-induced neurites are not equally susceptible to GRK2i and PMPMEase inhibitors (Figures 3B, C and 4B, C). Careful analysis indicates that while the percentage of cells bearing neurites was affected significantly in the presence of all three inhibitors, the average neurite lengths were modestly affected. It is likely that GRK2i or PMPMEase inhibitors inhibited the growing neurites and blocked neurite formation. On the other hand, inhibitors did not significantly affect longer neurites, which are relatively stable.

The dramatic rearrangement of MTs during neuronal differentiation is critical for vesicular transport, neurotransmitter release, and communication at synapse. Recent results suggest that Gβγ regulates the formation of SNARE complex, an essential step for neurotransmitter release of a synapse [60,61]. More recently, Gβγ has been shown to inhibit dopamine transporter activity [43]. Although it is not clear whether these events are interlinked, it is tempting to speculate that signals originating from cell-surface receptors utilize Gβγ to induce specific changes in MT assembly and organization in axons, which may in turn contribute to the Gβγ-dependent transport and neurotransmitter release of a synapse. Gβγ is known to activate a diverse array of effector molecules, including adenylate cyclases, phospholipases, PI3Kinase, and ion channels. Future investigation will be important to understand how these effector systems influence Gβγ-dependent regulation of MTs and neuronal differentiation. Recent results have indicated that MT assembly is severely compromised in the early stages of Alzheimer’s and Parkinson’s diseases [62-65]. Defects in MT-based transport is thought to be associated with many neurological disorders including Alzheimer’s disease, Huntington’s disease, and ALS [66-68] and disruption of the underlying microtubule network could be one way the transport is impaired [68]. We propose that the altered interaction of Gβγ with MTs may cause disruption of MTs and trigger an early stage of neurodegeneration. PMPMEase, which appears to regulate this interaction, may serve as a potential target for therapeutic intervention against neurodegenerative disorders.

## Conclusion

MTs play a key role in maintaining the highly asymmetric shape and structural polarity of neurons that are essential for neuronal functions. The process by which MT structure is remodeled in neurons is a central question in cell biology and our result suggests that Gβγ may play a role in this process. GPCRs as well as G protein subunits are abundant in neurons and have also been shown to regulate neurite outgrowth. The results presented here identify Gβγ as a potential key molecule in neurons that may utilize extracellular signals for the rearrangement of microtubules necessary for neuronal outgrowth and differentiation.

## Additional files

Additional file 1:
**Effect of preincubation of GRK2i on NGF-induced neuronal differentiation.** PC12 cells were pre-incubated with GRK2i for 2 h followed by 1-day treatment with NGF (100 ng/ml). The cells were then fixed and double labeled with anti-tubulin (red) and anti-Gβ (green) antibodies, and processed for confocal microscopy. Using Zeiss ZEN software, neurites were traced and measured, and the average neurite length and percent of cells bearing neurites were determined. **p* value < 0.05; ******p value* < 0.001 when compared to control. Additional file 2:
**Effect of PMPMEase inhibitors on preformed neurites.** PC12 cells were treated with 100 ng/mL of NGF for two consecutive days. Subsequently, cells were treated overnight with PMPMEase inhibitors, L-23 and L-28 (5 μM, and 10 μM), or the prototypical molecule PMSF (10 μM) and the cells were processed for confocal microscopy using anti-tubulin (red) and anti-Gβ (green) antibodies as described in the methods. Using Zeiss ZEN software, neurites were traced and measured, and the average neurite length and percent of cells bearing neurites were estimated. The differences between experimental conditions were assessed by one-way ANOVA. *p < 0.05 when compared to control or PMSF. Additional file 3:
**PC12 cells were treated overnight with PMPMEase inhibitors, L-23 and L-28 (5 μM, or 10 μM), or the prototypical molecule PMSF (10 μM) as indicated in the figure.** The cells were then fixed and double labeled with anti-tubulin (red) and anti-Gβ (green) antibodies and DAPI was used for nuclear staining (blue). Co-localization patterns are also shown in the merged images. PMSF did not seem to have any significant effect on organization of MT structure, Gβγ localization, and cellular morphology of PC12 cells (a–d). However, both L-23 and L-28 altered organization of the MTs and Gβγ similar to that observed in NGF-differentiated PC12 cells. Cellular aggregation was also evident in the presence of L-23 or L-28. Gβγ was concentrated in the cell-cell contact region in the presence of 10 μM L-28 and could be responsible for mediating cellular aggregation. Additional file 4:
**Co-localization of YFP-β1γ2 with MTs in PC12 cells overexpressing Gβγ.** The movie was generated by reconstructing high-resolution images using Volocity 3D Image Analysis software as indicated in the methods. Localization of overexpressed Gβγ (green) and its association with MTs (red) was clearly visible in the neurite by panning, zooming into, and rotating the 3-D image. Two cells are shown side by side, one with a long thin neurite, and the second cell with very short neurites. Both cells exhibit a similar labeling pattern. The movie shows that MTs and Gβγ interact throughout the neurite, as evidenced by clear yellow labeling. Gβγ labeling (green) was also observed alongside yellow labeling throughout the neuronal process, suggesting that Gβγ binds to MTs throughout the neurite.

## Abbreviations

MTs: Microtubules
ST: Soluble tubulin
MAP: Microtubule-associated protein
GPCR: G protein-coupled receptors
NGF: Nerve growth factor
GRK2: G protein-coupled receptor kinase 2
PMPMEase: Prenylated methylated protein methyl esterase
DMSO: Dimethyl sulfoxide
YFP: Yellow fluorescent protein
NGS: Normal goat serum
DNS: Differential nuclear staining
ROI: Region of interest
PMSF: Phenylmethylsulfonyl fluoride
