# Supplementary figures and images for: Nerve growth factor induces neurite outgrowth of PC12 cells by promoting Gβγ-microtubule interaction
Source: BMC Neurosci. 2014 Dec 31;15:132. doi: 10.1186/s12868-014-0132-4 (PMC4302597; doi:10.1186/s12868-014-0132-4)

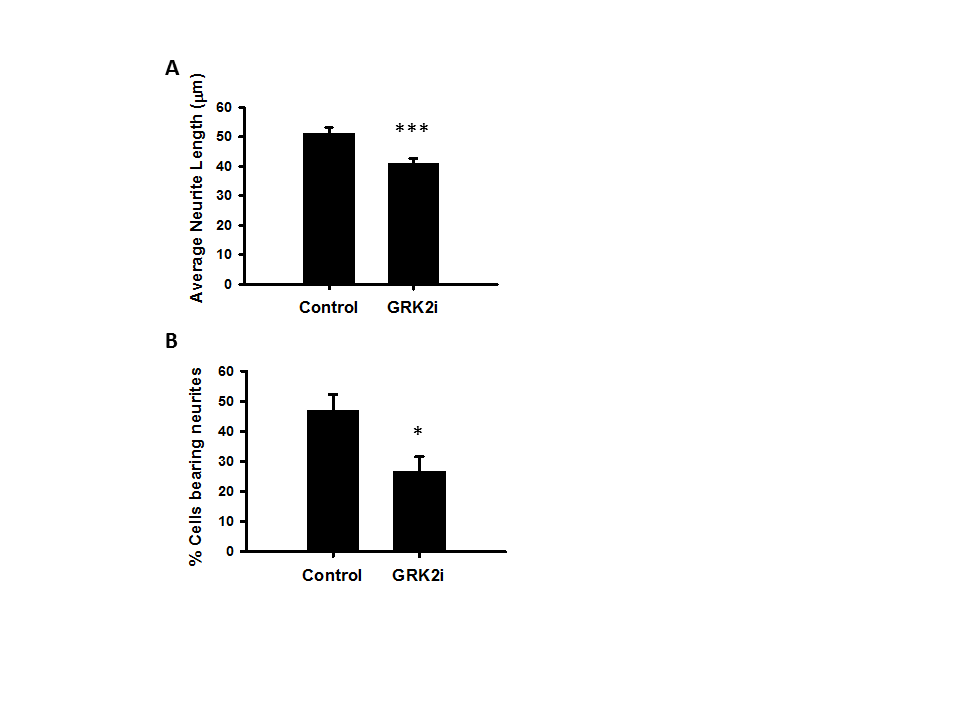

Supplement: Additional file 1: — Effect of preincubation of GRK2i on NGF-induced neuronal differentiation. PC12 cells were pre-incubated with GRK2i for 2 h followed by 1-day treatment with NGF (100 ng/ml). The cells were then fixed and double labeled with anti-tubulin (red) and anti-Gβ (green) antibodies, and processed for confocal microscopy. Using Zeiss ZEN software, neurites were traced and measured, and the average neurite length and percent of cells bearing neurites were determined. *p value < 0.05; ***p value < 0.001 when compared to control. [file 12868_2014_132_MOESM1_ESM.tiff]

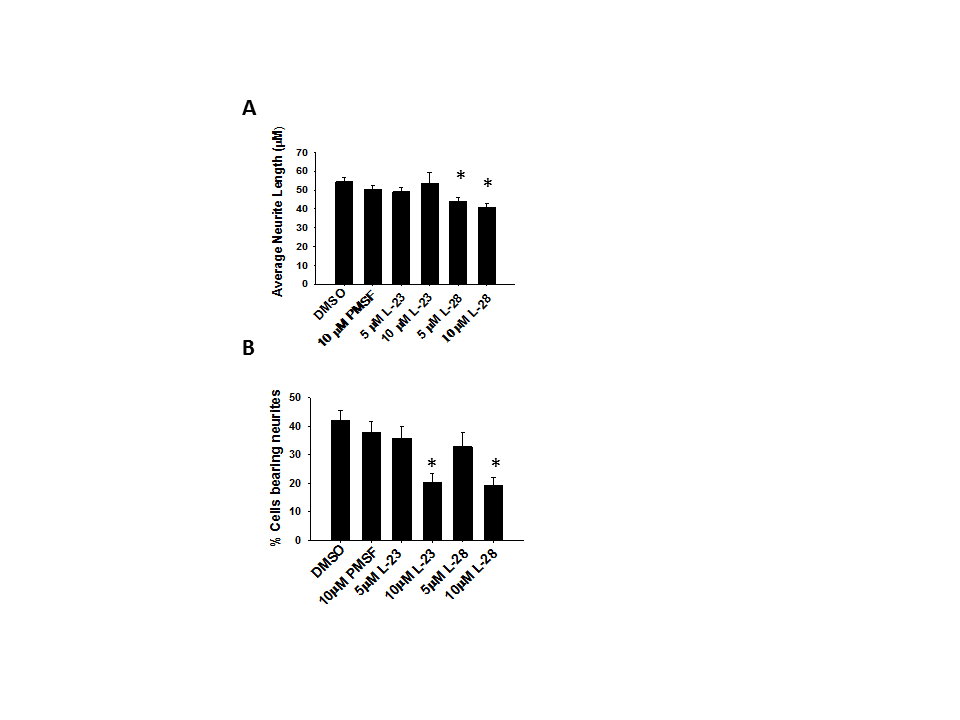

Supplement: Additional file 2: — Effect of PMPMEase inhibitors on preformed neurites. PC12 cells were treated with 100 ng/mL of NGF for two consecutive days. Subsequently, cells were treated overnight with PMPMEase inhibitors, L-23 and L-28 (5 μM, and 10 μM), or the prototypical molecule PMSF (10 μM) and the cells were processed for confocal microscopy using anti-tubulin (red) and anti-Gβ (green) antibodies as described in the methods. Using Zeiss ZEN software, neurites were traced and measured, and the average neurite length and percent of cells bearing neurites were estimated. The differences between experimental conditions were assessed by one-way ANOVA. *p < 0.05 when compared to control or PMSF. [file 12868_2014_132_MOESM2_ESM.tiff]

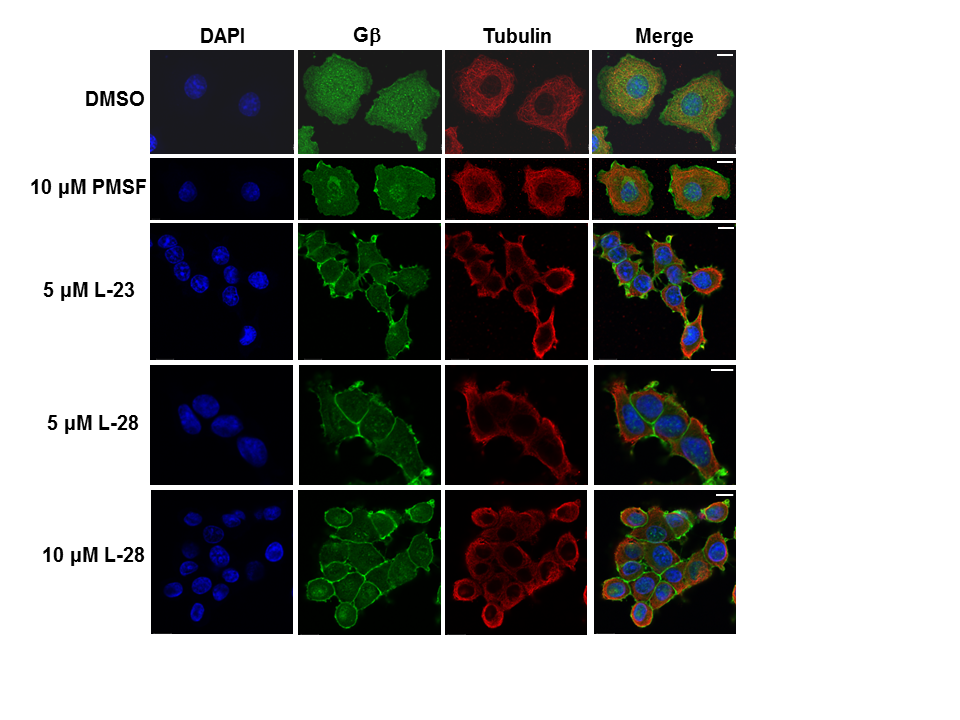

Supplement: Additional file 3: — PC12 cells were treated overnight with PMPMEase inhibitors, L-23 and L-28 (5 μM, or 10 μM), or the prototypical molecule PMSF (10 μM) as indicated in the figure. The cells were then fixed and double labeled with anti-tubulin (red) and anti-Gβ (green) antibodies and DAPI was used for nuclear staining (blue). Co-localization patterns are also shown in the merged images. PMSF did not seem to have any significant effect on organization of MT structure, Gβγ localization, and cellular morphology of PC12 cells (a–d). However, both L-23 and L-28 altered organization of the MTs and Gβγ similar to that observed in NGF-differentiated PC12 cells. Cellular aggregation was also evident in the presence of L-23 or L-28. Gβγ was concentrated in the cell-cell contact region in the presence of 10 μM L-28 and could be responsible for mediating cellular aggregation. [file 12868_2014_132_MOESM3_ESM.tiff]
